# Study on the Electro-Fenton Chemomechanical Removal Behavior in Single-Crystal GaN Pin–Disk Friction Wear Experiments

**DOI:** 10.3390/mi16020210

**Published:** 2025-02-12

**Authors:** Yangting Ou, Zhuoshan Shen, Juze Xie, Jisheng Pan

**Affiliations:** 1 School of Electromechanical Engineering, Guangdong University of Technology, Guangzhou 510006, China; 2 State Key Laboratory for High Performance Tools, Guangzhou 510006, China; 3 Guangzhou Communications Technician Institute, Guangzhou 510006, China

**Keywords:** single crystal GaN, chemical mechanical polishing, electro-Fenton, friction and wear

## Abstract

Electro-Fenton chemical mechanical polishing primarily regulates the generation of hydroxyl radicals (·OH) via the Fenton reaction through an applied electric field, which subsequently influences the formation and removal of the oxide layer on the workpiece surface, thereby impacting the overall polishing quality and rate. This study employs Pin–Disk friction and wear experiments to investigate the material removal behavior of single-crystal GaN during electro-Fenton chemical mechanical polishing. Utilizing a range of analytical techniques, including coefficient of friction (COF) curves, surface morphology assessments, cross-sectional analysis, and power spectral density (PSD) measurements on the workpiece surface, we examine the influence of abrasives, polishing pads, polishing pressure, and other parameters on the electro-Fenton chemical–mechanical material removal process. Furthermore, this research provides preliminary insights into the synergistic removal mechanisms associated with the electro-Fenton chemical–mechanical action in single-crystal GaN. The experimental results indicate that optimal mechanical removal occurs when using a W0.5 diamond at a concentration of 1.5 wt% combined with a urethane pad (SH-Q13K-600) under a pressure of 0.2242 MPa.

## 1. Introduction

Single-crystal GaN exhibits a broad spectrum of applications in short-wavelength light-emitting devices and high-power, high-frequency electronic systems, including blue–violet light-emitting diodes [1,2] and laser diodes [3]. This is attributed to its remarkable physical and chemical properties, such as a direct wide bandgap, a high breakdown electric field, enhanced thermal stability, and exceptional chemical inertness. Furthermore, single-crystal GaN-based micro-sized diodes have significantly advanced the rapid evolution of the micro-self-emitting display industry, which holds considerable promise for future developments in virtual reality (VR) technology [4,5], augmented reality (AR) technology [6,7], and biomedical probes [8,9].

Due to the excellent thermal stability and chemical inertness of single-crystal GaN, traditional surface polishing methods yield suboptimal efficiency on GaN surfaces. Furthermore, the use of hard abrasives for mechanical polishing often results in subsurface damage within the polished workpieces, adversely affecting the optical and electrical properties of single-crystal GaN [10]. To achieve enhanced polishing efficiency and superior surface quality, numerous chemical mechanical polishing (CMP) assisted techniques have been explored by researchers. Methods such as optical-assisted CMP [11,12], electrochemical-assisted CMP [13,14], Fenton-assisted CMP [15,16], and electro-Fenton-assisted CMP [17,18] have gained widespread acceptance in the polishing of single-crystal GaN. Electro-Fenton CMP [19] primarily involves two processes: firstly, hydroxyl radicals (·OH) generated by the electro-Fenton reaction facilitate the formation of Ga_2_O_3_ with a lower hardness on the workpiece surface; secondly, this oxidized layer is subsequently removed through mechanical action. In this context, the reduction reactions occurring within the electric field effectively enhance the conversion rates of Fe^2+^ and Fe^3+^ ions, thereby promoting ·OH generation and facilitating oxidation reactions on the workpiece surface. This mechanism serves to minimize subsurface damage following polishing while improving both polishing quality and material removal capability efficiency [20]. Moreover, during electro-Fenton CMP, the rates of Ga_2_O_3_ generation and removal from the oxide layer significantly influence polishing quality. To achieve a synergistic effect between the chemical and mechanical actions in this process, it is essential to investigate its material removal behavior. Extensive research has been conducted by scholars regarding the material removal mechanisms associated with synergistic chemical and mechanical actions in CMP polishing. Zhang et al. [21] investigated the chemical–mechanical synergy (CMS) of the TiO_2_-catalyzed H_2_O_2_ synergistic removal of single-crystal 6H-SiC under UV irradiation through friction wear analysis. The results indicated that anatase TiO_2_ exhibited both photocatalytic and abrasive effects during friction under UV light, encompassing mechanisms such as repair, rolling, plowing, polishing, and protective film formation. They subsequently modeled the TiO_2_-catalyzed removal of H_2_O_2_ from single-crystal SiC materials in UV conditions and visualized the synergistic interactions between the mechanical and chemical processes. Guo et al. [22] employed atomic force microscopy to perform nano-wear tests on calcium fluoride (CaF_2_) crystals using SiO_2_ microsphere tips across various pH solutions to investigate the atom removal mechanism in CMP. Their findings revealed no significant subsurface damage upon transmission electron microscopy (TEM) analysis of wear trajectories, thereby confirming the predominant role of friction-induced chemical reactions in this process. Pan et al. [17] explored the friction wear behavior of single-crystalline GaN enhanced by electro-Fenton chemistry through ball–disk friction experiments; their results demonstrated that the electro-Fenton medium provided optimal oxidation and corrosion effects on the GaN surface, elucidating the synergistic mechanism underlying chemical and mechanical material removal during frictional wear processes. Vladimir Totolin et al. [23] utilized COF curves and wear area measurements to evaluate the material removal capability of GaN. The results indicated that both the average COF and wear area of GaN exhibited an increasing trend with enhanced oxidation by the surrounding medium, thereby improving the removal performance of GaN surface materials.

In this study, Pin–Disk friction and wear experiments were employed to simulate the contact and material removal interactions between the polishing pad, abrasive, and workpiece during electro-Fenton chemical–mechanical polishing. A variety of testing methodologies were utilized, including COF curves, surface morphology analysis, cross-sectional profiling, and PSD assessments. These methods aimed to investigate how factors such as abrasives, polishing pads, and the applied pressure influence material removal efficiency and oxide layer formation on the workpiece surface. Furthermore, this research seeks to elucidate the synergistic removal mechanisms associated with the electro-Fenton chemical–mechanical action on single-crystal GaN, thereby advancing the electro-Fenton chemical–mechanical polishing technology.

## 2. Experimental Materials and Principles

### 2.1. Experimental Materials

**Figure 1:**
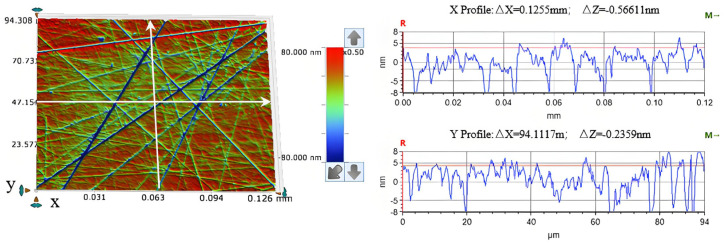
Surface morphology of the GaN crystal wafer before wear. (Note: The three arrows above and below the height scale in the figure are merely the zoom-in and zoom-out indicators of the detection software and do not affect the content of the article).

The reagents utilized in this experiment included: H_2_O_2_ (AR, 30 wt%), diamond abrasive (particle size: 1 μm, 99 wt%, Zhengzhou Xingke Superhard Material Co., Ltd., Zhengzhou, China), Al_2_O_3_ abrasive (particle size: 1 μm; 99 wt%, Longao Trading Co., Ltd., Nangong City, China (NCLT)), SiO_2_ abrasive (particle size: 1 μm, 99 wt%, NCLT), silicon carbide abrasive (particle size: 1 μm, NCLT), anhydrous sodium sulfate (AR, 99 wt%), citric acid (AR, 99 wt%), anhydrous ethanol (AR, 99 wt%), multi-walled carbon nanotubes MWCNT (Shenzhen Turing Artificial Intelligence Black Technology Co., Ltd., Shenzhen, China), Fe-C filler (particle size 2~4 mm, Green Plains Activated Carbon Co., Ltd., Pingdingshan City, China), the cathode material is graphite felt (Shanghai Carbon Factory, Shanghai, China), and the anode material is nickel foam (Wuzhong Economic Development Zone, Chengnan Jinyihe New Materials Operation Department, Suzhou, China). All types of polishing pads used in the experiment were provided by Xinpade Optoelectronic Technology Co., Ltd. in Dongguan City, China. The polishing test samples were 10 mm × 10 mm single-crystal GaN wafers. To minimize test errors, the single-crystal GaN workpiece was uniformly ground before the experiment to ensure consistent surface roughness parameters: R_a_ ≈ 3 nm and R_q_ ≈ 5 nm. The surface morphology after grinding is shown in Figure 1.

### 2.2. Experimental Principles and Characterization Methods

**Figure 2:**
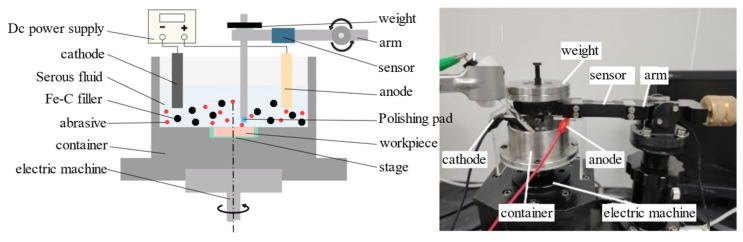
Experimental device and schematic diagram of Pin–Disk friction and wear.

The friction and wear experiments utilizing Pin–Disk configurations were conducted to simulate the contact and material removal mechanisms between the polishing pad, abrasive, and workpiece in the electro-Fenton chemical–mechanical polishing process. In these experiments, the polishing pad is embedded in the friction pin and fixed on the support arm. The container fixture is mounted on the carrier table, enabling the container to rotate with the spindle at a specified speed and injected with an electro-Fenton chemical mechanical polishing solution. In this process, the contact formed between the polishing pad–abrasive grains–workpiece and polishing pad–workpiece are established, and the schematic diagram of the Pin–Disk friction wear experiment is shown in Figure 2.

The experimental apparatus employed was the WTM-2E-controlled atmosphere micro-friction and wear tester (see Figure 2). Before the experiment, the support arm was calibrated to a horizontal orientation using the elevation screw, while the experimental friction radius was set by adjusting the forward and backward movement screws. During experimentation, the workpiece was securely affixed to the container, which was rotated at a predetermined speed by a servo motor. The sensor mounted on the support arm converts the frictional force generated during motion into an electrical signal, which is automatically recorded by a computer and subsequently output as a friction coefficient curve.

By analyzing the trends in the COF curve and the surface scratching characteristics, this study evaluates the impact of various parameter conditions on the surface effects of workpieces subjected to chemical and mechanical removal processes. Building upon these findings, further exploration and validation of the synergistic interactions between these two mechanisms are conducted. The COF serves as an indicator of the contact state and area among the polishing pad, abrasive, and workpiece [24]; notably, a higher COF correlates with an increased material removal rate from the workpiece surface [25]. Furthermore, material removal from the workpiece surface facilitates oxide layer formation while simultaneously accelerating its elimination. Consequently, we employed a WTM-2E-controlled atmosphere micro-friction and wear tester for the real-time monitoring of COF to investigate how varying parameters influence material–surface contact states. Additionally, average COF values were utilized to assess the effectiveness of material removal and oxide layer generation under different conditions. Surface morphology, cross-sectional profiles, and PSD power density characteristics post-friction were analyzed using a BRUKER ContourGT-X white light interferometer. PSD allows for detailed analysis within specific spatial frequency ranges. According to Parseval’s theorem, larger PSD values within designated frequency bands indicate greater surface roughness.

Within a specific frequency range, a correlation exists among the root mean square deviation of the surface profile (R_q_), the arithmetic average deviation of the surface profile (R_a_) with respect to the PSD curve, as illustrated in Equations (1) and (2). In other words, within this defined frequency range, an increase in the PSD value corresponds to a greater surface roughness (R_a_), indicating that the workpiece surface becomes progressively rougher.

(1) $$R_{q}=\sqrt{\sum_{{\upsilon =\upsilon }_{1}}^{\upsilon_{2}}\mathrm{PSD}(\upsilon )\Delta \upsilon }$$

(2) $$R_{a}=\frac{2\sqrt{2}}{\pi }R_{q}$$

Before and after the experiment, the BRUKER Contour GT-X white light interferometer was used to examine the surface of single-crystal GaN. Subsequently, surface profiles and cross-sectional curves of the workpiece were generated using Vision 2015 software. The three-dimensional surface morphology allows for direct observation of scratches on the workpiece surface, while the cross-sectional curves provide insights into both the depth and distribution of these scratches. For the surface PSD analysis, we employed Vision software to perform 2D XY-averaged PSD analysis, thoroughly investigating the effects of various factors on the frequency characteristics and surface morphology of the workpiece.

### 2.3. Experimental Method and Design

In this study, the contact and removal processes between the polishing pad, abrasive, and workpiece during electro-Fenton chemical mechanical polishing were simulated using a Pin–Disk friction and wear experiment. The specific experimental procedures are outlined as follows:

(1) Preparation of the electro-Fenton solution: A specified amount of anhydrous Na_2_SO_4_ electrolyte was dissolved in deionized water, followed by the addition of citric acid to adjust the pH to 3. The solution was mixed uniformly under ultrasonic agitation before incorporating a designated quantity of Fe-C Fenton catalyst for subsequent use.
(2) Setup and parameter configuration of the apparatus: The pre-treated single-crystal GaN was securely mounted in a container, which was then placed on the load-bearing platform of the apparatus. The support arm was adjusted to a horizontal position using the elevation stage, while the experimental friction radius was set using the forward-and-backward movement knob, which was subsequently tightened. Electrodes were installed, with modified amino-MWCNT graphite felt serving as the cathode and foam nickel as the anode. Relevant experimental parameters were configured, including dynamic zeroing before equipment activation. Files were saved after stopping rotation before disassembling the workpiece.
(3) Cleaning procedure for the workpiece: The disassembled workpiece underwent ultrasonic cleaning in isopropyl alcohol for one minute and was marked for subsequent analysis.

The fundamental parameters related to the electro-Fenton solution preparation along with the basic experimental conditions for the Pin-Disk simulated CMP friction and wear experiments are presented in Table 1, while details regarding the experimental design can be found in Table 2.

### 2.4. Experimental Feasibility Verification

**Figure 3:**
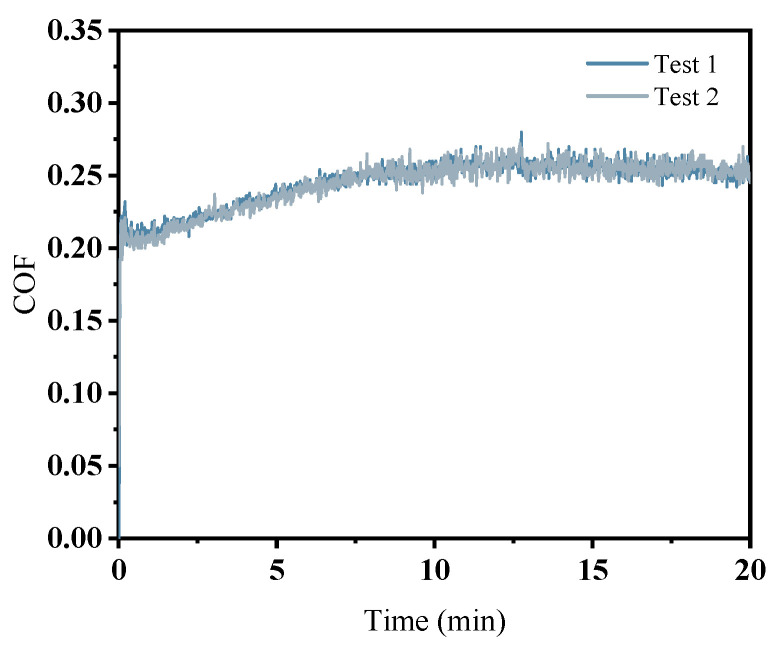
Repeated Test Friction Coefficient.

Fluctuations in the COF are observed during friction and wear processes. To address the uncertainties inherent in the testing procedure, two experiments were conducted under electrolyte conditions with a pH of 3 (A1). As illustrated in Figure 3, the COF variations from both tests demonstrate strong consistency. In both cases, COFs increased rapidly within the first minute, reaching a peak value of 0.23 before experiencing a rapid decline. Subsequently, they began to gradually rise and stabilize after 0.5 min. The similar trends observed in the COF changes across both tests suggest that repeating the experiments can effectively mitigate uncertainty in friction and wear test outcomes.

## 3. Experimental Results and Discussion

### 3.1. Effect of Abrasive Type on the Removal Behavior of Single-Crystal GaN Materials

In the electro-Fenton chemical mechanical polishing liquid system, the influence of various abrasive materials on the material removal capability and the promotion of oxide layer formation on the workpiece surface was investigated through Pin–Disk wear experiments utilizing different abrasives (diamond W1, silicon carbide W1, aluminum oxide W1, and silica sol 120 nm).

**Figure 4:**
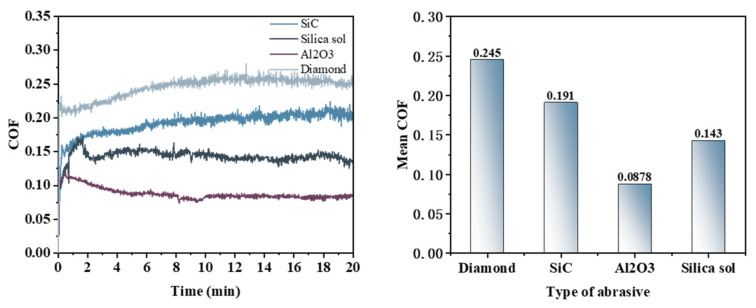
Friction coefficient curves and average friction coefficient under different abrasive conditions.

Figure 4 illustrates that the trends in friction coefficient curves vary among the different abrasives used in the Pin–Disk wear experiment. When diamond is employed as the abrasive, the COF increases rapidly within a short duration before exhibiting a gradual growth trend; it peaks at approximately 11 min and subsequently stabilizes. In contrast, when silicon carbide serves as the abrasive, the COF quickly reaches a stable value, with an average COF of 0.191—lower than that observed with diamond (0.245). Both silica sol and alumina exhibit a rapid initial increase in COF followed by a gradual decline; notably, when alumina is utilized as an abrasive, this decrease is more pronounced. The COF reflects the material removal capability of both the polishing pad and the abrasive on the workpiece surface: higher COF values correspond to increased material removal rates [25,26]. The average friction coefficients for various abrasives are ranked as follows: 0.245 (diamond W1) > 0.191 (silicon carbide W1) > 0.143 (silica sol 120 nm) > 0.0878 (alumina W1). Thus, within the electro-Fenton chemical mechanical polishing system, the capacity to remove oxide layers from workpiece surfaces decreases in order from diamond W1 to silicon carbide W1 to silica sol 120 nm and finally alumina W1.

**Figure 5:**
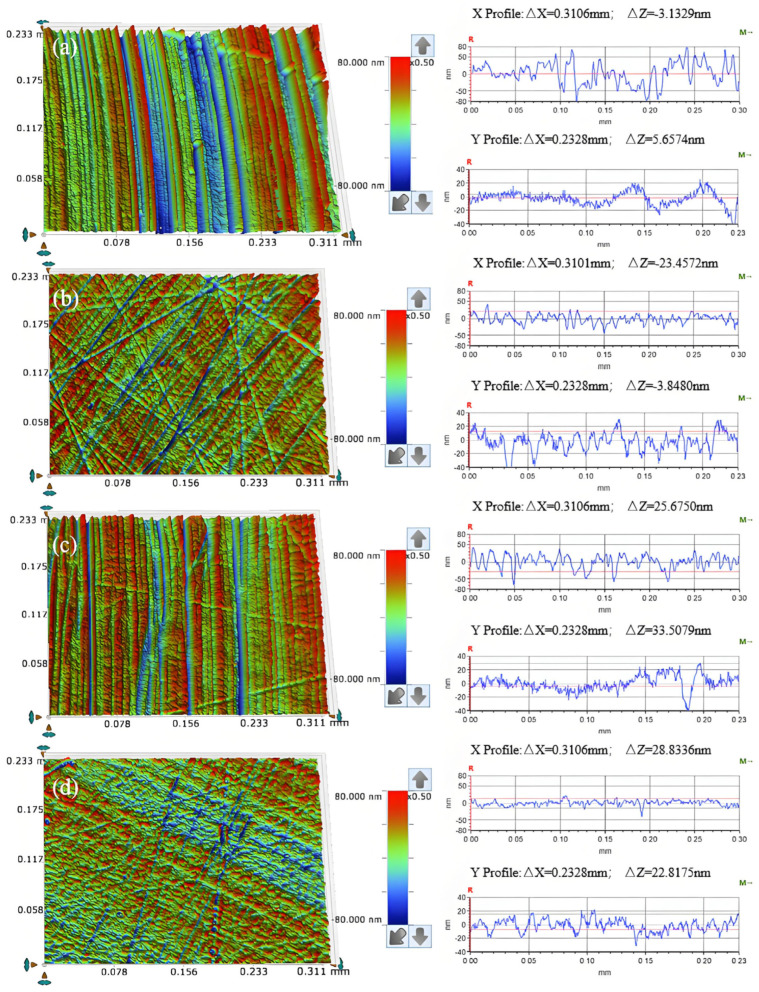
Surface morphology (**left**) and cross section curve (**right**) of friction and wear on the workpiece under different abrasive conditions (**a**) W1 diamond; (**b**) W1 silicon carbide; (**c**) W1 aluminum oxide; (**d**) 120 nm silica sol.

As illustrated in Figure 5a, uniform scratches are observed on the surface of the workpiece following a period of chemical–mechanical removal when diamond is employed as the abrasive; in Figure 5b, when silicon carbide serves as the abrasive, messy scratches appear on the workpiece surface after a similar duration of chemical–mechanical removal, accompanied by localized fluctuations in the cross-sectional curve. Figure 5c demonstrates that, with aluminum oxide as the abrasive, irregular scratches are evident on the workpiece surface after this process, along with significant fluctuations in specific areas of the cross-sectional curve. Finally, Figure 5d reveals that when silica sol is utilized as an abrasive, noticeable scratches remain on the workpiece surface post-chemical and mechanical removal, as evidenced by surface morphology and cross-sectional analysis.

**Figure 6:**
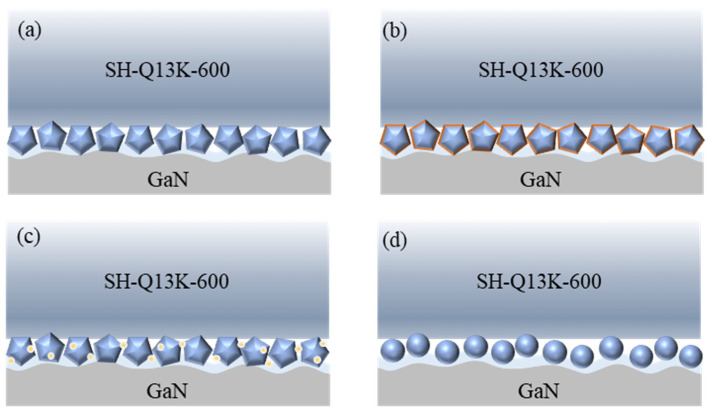
Friction wear material removal behavior under different abrasive conditions (**a**) W1 diamond; (**b**) W1 silicon carbide; (**c**) W1 aluminum oxide; (**d**) 120 nm silica sol.

In the same electro-Fenton system, the chemical nature and structural properties of the abrasive species will significantly affect the material removal behavior of the Pin–Disk friction wear on the workpiece surface. As shown in Figure 6a, diamond abrasives are chemically stable and hard and can be uniformly dispersed in the polishing solution during friction wear, indicating that diamond abrasives have a stable removal effect on the oxide layer. While removing the surface of the workpiece, the abrasive promotes the formation of the oxide layer [27], thus achieving a stable alternation between chemical action and mechanical removal, and achieving a better material removal effect [28]. As shown in Figure 6b, the silicon carbide abrasives can be oxidized by ·OH radicals generated by the electro-Fenton process to form oxides with a lower hardness [29] which reduces the hardness of the abrasive surface, and the generated oxides may act as a lubricant between the polishing pad and the workpiece during friction, which reduces the material removal rate. In addition, the reduction in effective ·OH radicals acting on the workpiece surface leads to a decrease in the thickness of the oxide layer formed, which in turn reduces the amount of abrasive removal from the surface of the workpiece. As shown in Figure 6c, unlike silicon carbide, alumina can generate ·OH [30] catalyzed by H_2_O_2_, which leads to a reduction in the effective abrasive action on the oxide layer on the surface of the workpiece, resulting in a significant reduction in the removal of alumina from the surface of the workpiece. As shown in Figure 6d, silica sol has a low hardness and a spherical shape, making it produce rolling friction in the friction process, so the material removal ability is lower.

**Figure 7:**
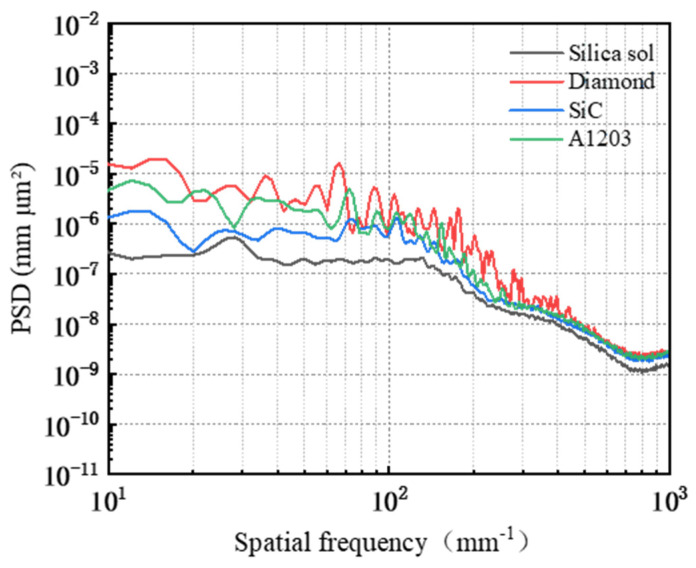
Surface power spectral density distribution under different abrasive conditions.

The PSD analysis was performed on the worn surface of the workpiece following friction and wear testing, with the results presented in Figure 7. In the frequency range from 10 mm^−1^ to 70.32 mm^−1^, the PSD curves for the diamond and alumina abrasives exhibit overlap, while those for silica sol, alumina, and silicon carbide show a distinct hierarchical pattern, arranged from top to bottom as alumina, silicon carbide, and silica sol. Within the frequency range from 70.32 mm^−1^ to 452.07 mm^−1^, the PSD curves for silicon carbide and alumina intersect but do not coincide; their corresponding order of PSD values is diamond > alumina > silicon carbide > silica sol. Beyond 452.07 mm^−1^, all four abrasive types display a gradual decline in their PSD curves that tend toward stabilization. Notably, the PSD curves for diamond, alumina, and silicon carbide tend to converge; in contrast to these three abrasives’ values mentioned earlier, that of silica sol remains relatively lower. Thus, it can be inferred that within this frequency range, the three-dimensional surface morphology of workpieces associated with diamond, silicon carbide, and alumina abrasives exhibit similarity.

Integrating the surface morphology images, cross-sectional curves, and COF analysis results from the friction and wear experiments allows for the conclusion that the material removal capability of different abrasives follows this order: diamond > silicon carbide > silica sol > aluminum oxide. Specifically, when diamond is employed as the abrasive, the chemical reactions within the system effectively facilitate oxide layer formation, which can subsequently be removed promptly from the workpiece surface.

### 3.2. Effect of Abrasive Concentration on the Removal Behavior of Single-Crystal GaN Materials

In the electro-Fenton chemical mechanical polishing system, the influence of varying concentrations (0.5 wt%, 1 wt%, 1.5 wt%, and 2 wt%) of W1 diamond abrasive on the material removal capability and promotion of oxide layer formation on the workpiece surface was investigated through Pin–Disk friction wear experiments.

**Figure 8:**
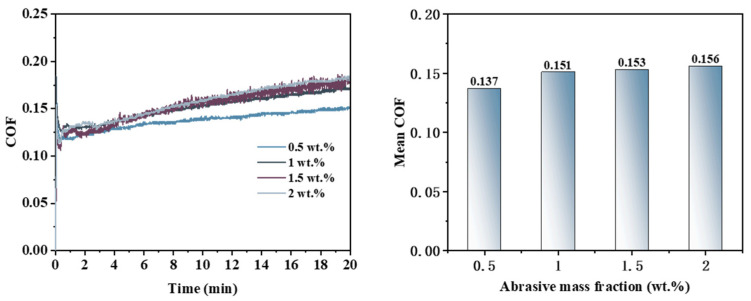
Friction coefficient curve and average friction coefficient under different abrasive concentration conditions.

As illustrated in Figure 8, during the Pin–Disk friction wear experiments, the friction coefficient curve exhibits a consistent trend when utilizing the same type of abrasive at varying concentrations: the COF reaches a specific value within a short duration and subsequently increases gradually. With extended reaction times and higher abrasive concentrations, both the magnitude and rate of the COF increase also become more pronounced. The average friction coefficients, ranked from highest to lowest, are as follows: 0.156 (2 wt%) > 0.153 (1.5 wt%) > 0.151 (1 wt%) > 0.137 (0.5 wt%). This indicates that an increase in the abrasive concentration correlates with a gradual rise in the average COF.

**Figure 9:**
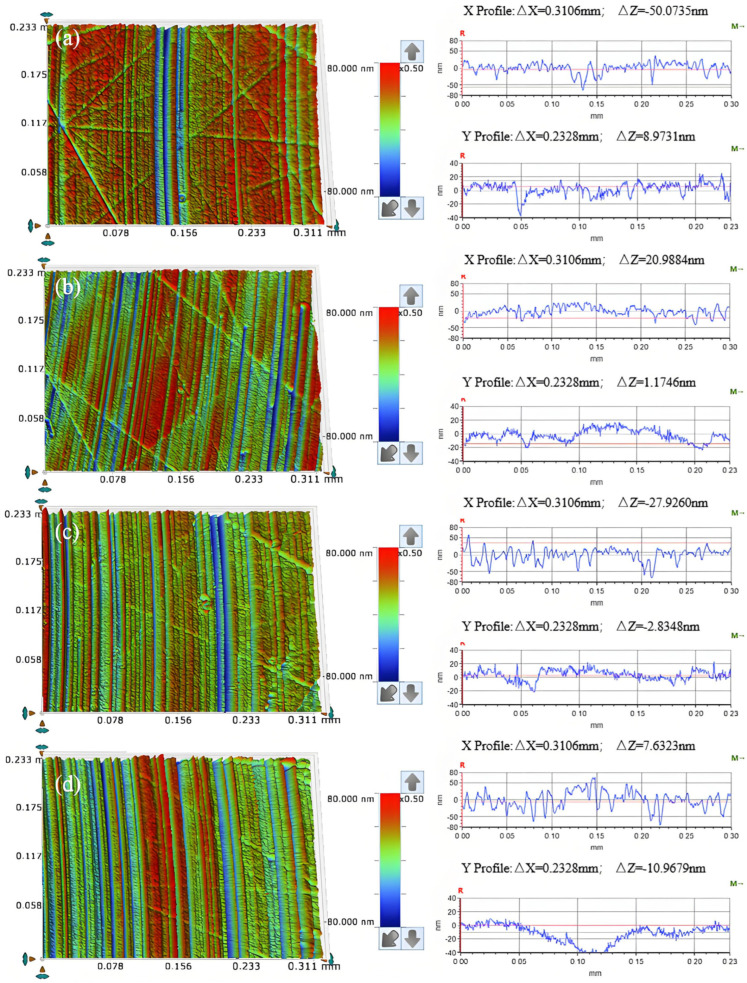
Surface morphology (**left**) and cross-section curves (**right**) of friction wear workpieces under different abrasive concentration conditions (**a**) 0.5 wt%; (**b**) 1 wt%; (**c**) 1.5 wt%; (**d**) 2 wt%.

As illustrated in Figure 9a, at an abrasive concentration of 0.5 wt%, the workpiece surface exhibits a regular scratch of material removal, accompanied by noticeable random scratches. The cross-sectional curve reveals significant fluctuations in both the X and Y directions. In Figure 9b, with an abrasive concentration of 1 wt%, the post-friction and wear surface displays slightly more regular scratches, although some random scratches remain evident. Upon increasing the concentration to 1.5 wt% (Figure 9c), irregular scratches on the workpiece surface are markedly reduced, predominantly presenting as regular scratches. In Figure 9d, at a concentration of 2 wt% abrasive, these regular scratches become even more pronounced. However, analysis of the cross-sectional curve indicates that fluctuations perpendicular to the direction of regular scratches (X Profile) are prominent, while the material removal depth shows a trend from shallow to deep along the direction of these regular scratches (Y Profile).

**Figure 10:**
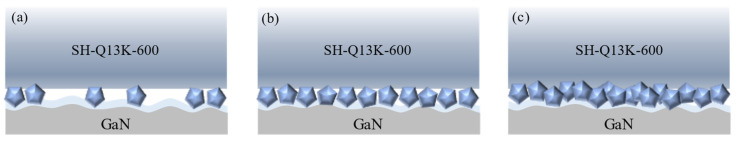
Friction wear material removal behavior under different abrasive concentration conditions. (**a**) the concentration of abrasive is relatively low; (**b**) the abrasive concentration is moderate.; (**c**) the concentration of abrasive is relatively high.

In the Pin–Disk friction wear process, the concentration of abrasive particles is intricately linked to the material removal dynamics on the workpiece surface, as illustrated in Figure 10. At low concentrations of abrasive particles, their limited presence on the workpiece surface results in insufficient oxide layer removal, leaving behind initial rough scratches. As the concentration increases, a greater number of abrasive particles interact with the workpiece surface, thereby enhancing oxide layer removal and facilitating the formation of new oxide layers. With further increases in abrasive concentration, a notable reduction in residual rough scratches on the workpiece surface occurs. However, as more abrasive particles act upon the surface, a continuous decrease in pressure leads to diminished depth penetration of these particles into the oxide layer. This ultimately renders material removal actions unstable and reduces overall removal efficiency.

**Figure 11:**
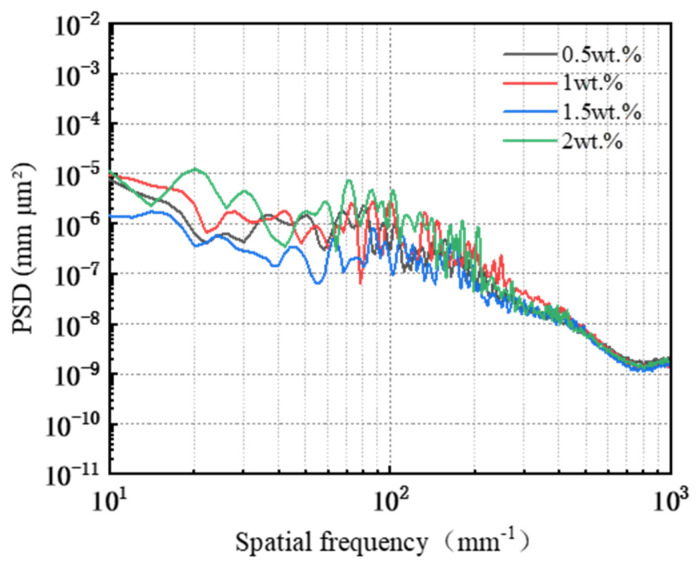
Surface power spectral density distribution under different abrasive concentrations.

A PSD analysis was performed on the surface of the workpiece following friction and wear, with the results presented in Figure 11. In the frequency range from 10 mm^−1^ to 62.29 mm^−1^, the PSD curves corresponding to three different abrasive concentrations (0.5 wt%, 1 wt%, and 2 wt%) exhibit overlap without coinciding, while the PSD value for the 1.5 wt% concentration is significantly lower than those of the other three concentrations. Within the frequency range from 62.29 mm^−1^ to 389.78 mm^−1^, the PSD curves corresponding to the four abrasive concentrations cross but do not overlap each other. Beyond a frequency of 389.78 mm^−1^, these curves converge and display a gradual decline before leveling off, indicating that the surface topography remains similar across all four abrasives within this range. Consequently, it can be inferred that the variations in surface roughness Ra when utilizing different abrasive concentrations are primarily concentrated within two specific frequency ranges: from 10 mm^−1^ to 62.29 mm^−1^ and from 62.29 mm^−1^ to 389.78 mm^−1^.

Integrating the surface morphology images, cross-sectional curves, and COF analysis results obtained from the friction and wear experiments, it can be inferred that the effectiveness of material removal at varying abrasive concentrations is ranked as follows: 2 wt% > 1.5 wt% > 1 wt% > 0.5 wt%. Conversely, the surface quality of the workpiece is ordered from best to worst as follows: 1.5 wt% > 2 wt% > 1 wt% > 0.5 wt%.

### 3.3. Effect of Abrasive Grain Size on the Removal Behavior of Single-Crystal GaN Materials

In the electro-Fenton chemical mechanical polishing system, various sizes of diamond abrasives (W0.2, W0.5, W1, W1.5) were employed in Pin–Disk wear tests to examine the effect of abrasive particle size on both the material removal efficiency and the promotion of oxide layer formation on the workpiece surface.

**Figure 12:**
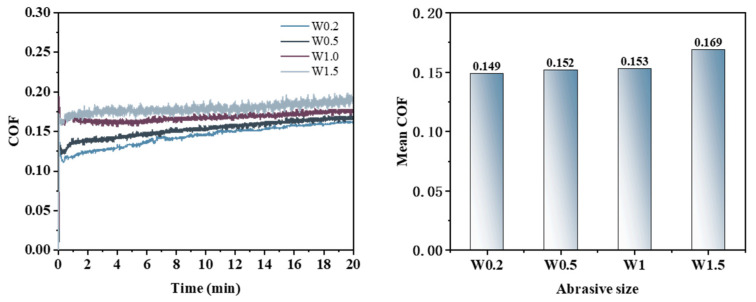
Friction coefficient curve and average friction coefficient under different abrasive particle size conditions.

Figure 12 illustrates that the growth rate and amplitude of the friction coefficient vary with different abrasive particle sizes, and the patterns of change in the friction coefficient curves differ accordingly. For abrasive particle sizes W0.2 and W0.5, the COF reaches a peak value within a short duration, followed by a gradual increase. In contrast, for particle sizes W1 and W1.5, the COF stabilizes after reaching a certain value quickly. The average friction coefficients ranked from highest to lowest are as follows: 0.169 (diamond W1.5) > 0.153 (diamond W1) > 0.152 (diamond W0.5) > 0.149 (diamond W0.2).

At smaller abrasive particle sizes, surface scratches tend to be relatively narrow and dense, thereby increasing the area of the workpiece exposed to the electro-Fenton chemical mechanical polishing solution; consequently, over time, the abrasives enhance their effectiveness in removing oxide layers from the workpiece surface. However, as abrasive particle size increases further, larger particles can penetrate more deeply into the workpiece surface and remove oxide layers more effectively; thus, an increase in the average COF is observed with larger abrasive particle sizes.

**Figure 13:**
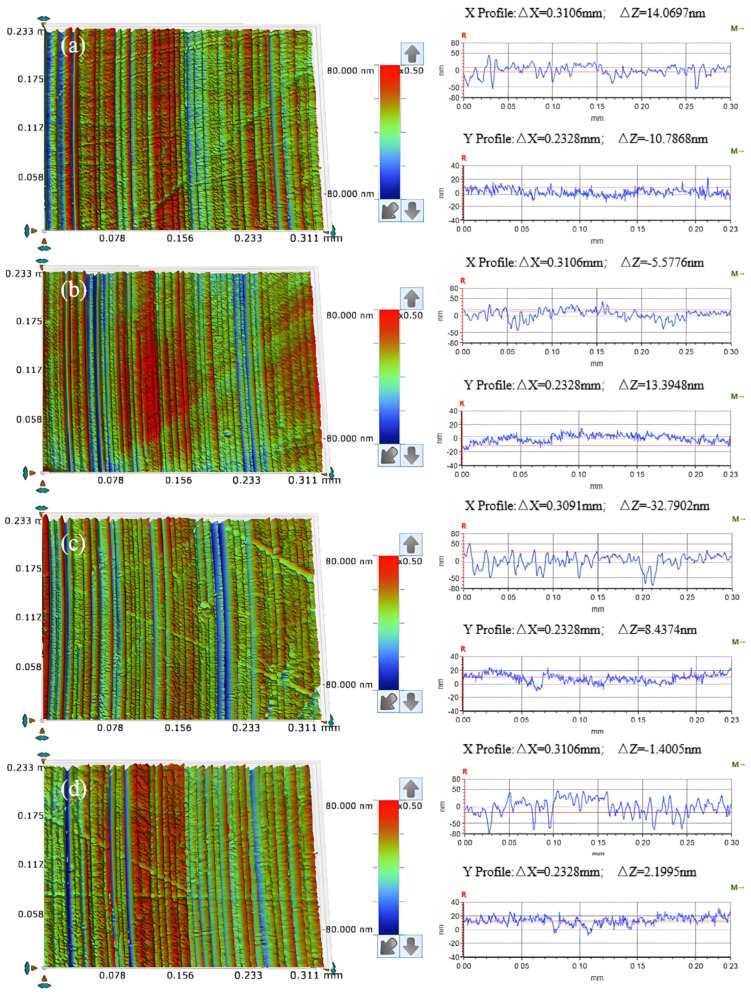
Surface morphology (**left**) and cross-section curves (**right**) of friction wear workpieces under different abrasive grain size conditions (**a**) W0.2; (**b**) W0.5; (**c**) W1; (**d**) W1.5.

Figure 13a illustrates that when the abrasive particle size is W0.2, the surface quality of the workpiece after frictional wear exhibits a regular pattern of scratches, with the cross-sectional curve displaying relatively gentle variations; however, some chaotic scratches remain present. In Figure 13b, for an abrasive particle size of W0.5, the surface quality continues to show a regular pattern of scratches without any chaotic cutting, and the cross-sectional curve remains smooth. As depicted in Figure 13c, when the abrasive particle size is W1, the workpiece’s surface quality retains a regular scratch pattern, although certain scratches are notably deeper and exhibit localized fluctuations in the cross-sectional curve. Finally, for an abrasive particle size of W1.5, as shown in Figure 13d, there exists a consistent scratch pattern on the workpiece surface post-frictional wear, with some scratches and fluctuations in the cross-sectional curve being even more pronounced.

**Figure 14:**
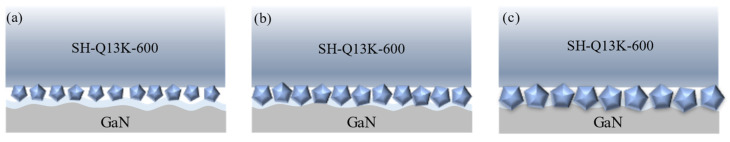
Material removal behavior under different abrasive grain size conditions (**a**) the abrasive particle size is smaller; (**b**) the abrasive particle size is smaller; (**c**) the abrasive particle size is too large.

Under the same conditions, the size of the abrasive grain significantly influences the depth of its embedding in the oxide layer on the surface of the workpiece. Smaller abrasive grain sizes tend to embed less deeply into this oxide layer, thereby diminishing their effectiveness in removing it. As illustrated in Figure 14, an increase in the abrasive grain size results in larger particles embedding more profoundly into the surface oxide layer, facilitating the complete removal of the generated oxide and leading to a uniform and regular scratch pattern on the workpiece surface. Furthermore, as abrasive grain size continues to increase, so does the depth of embedding, which not only removes the surface oxide layer but may also reach the unoxidized material beneath, resulting in pronounced deep scratches on the workpiece surface.

**Figure 15:**
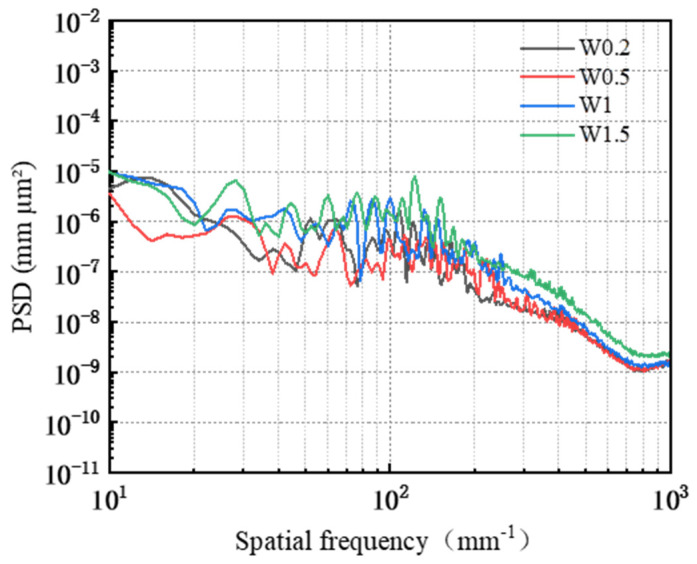
Surface power spectral density distribution under different abrasive grain size conditions.

The PSD analysis was performed on the surface of the worn workpiece, with the results presented in Figure 15. In the frequency range from 10 mm^−1^ to 24.11 mm^−1^, the PSD curves for abrasive grain sizes W0.2, W1, and W1.5 exhibit overlap without coinciding; notably, the PSD value for abrasive grain size W0.5 is significantly lower than those corresponding to the other three sizes. Within the frequency range from 24.11 mm^−1^ to 277.27 mm^−1^, the PSD curves corresponding to the four abrasive grain sizes intersected but did not overlap, with values ranked in descending order as follows: W1.5 > W1 > W0.2 > W0.5. Beyond a frequency of 277.27 mm^−1^, the PSD curves for four abrasive grain sizes demonstrate a gradual decline and approach a plateau; specifically, those for abrasives W0.2 and W0.5 converge closely, indicating that their corresponding three-dimensional surface morphologies are similar on the workpiece surface. In contrast to these two sizes’ PSD values, those for abrasives W1 and W1.5 are larger overall; thus, within this frequency range, they rank as follows: W1.5 > W1 > W0.2 > W0.5.

Integrating the surface morphology images, cross-sectional curves, and COF analysis results obtained from the friction and wear experiments, it can be concluded that under varying abrasive grain size conditions, the effectiveness of material removal is ranked as follows: W1.5 > W1 > W0.5 > W0.2. Conversely, the surface quality of the workpiece ranks from highest to lowest as follows: W0.5 > W0.2 > W1 > W1.5.

### 3.4. Effect of Polishing Pad Type on the Removal Behavior of Single-Crystal GaN Materials

The various types and associated parameters of polishing pads are presented in Table 3. This study conducts friction and wear experiments using different polyurethane polishing pad types to investigate their influence on the material removal capability and the promotion of oxide layer formation on the workpiece surface.

**Figure 16:**
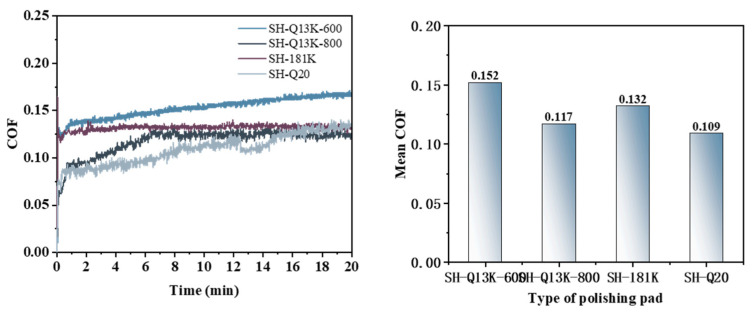
Friction coefficient curves and average friction coefficients for different polishing pad conditions.

Figure 16 illustrates that the friction coefficient curves from the Pin–Disk wear tests utilizing different types of polishing pads exhibit distinct trends. The average friction coefficients, ranked from highest to lowest, are as follows: 0.152 (SH-Q13K-600) > 0.132 (SH-181K) > 0.117 (SH-Q13K-800) > 0.109 (SH-Q20).

**Figure 17:**
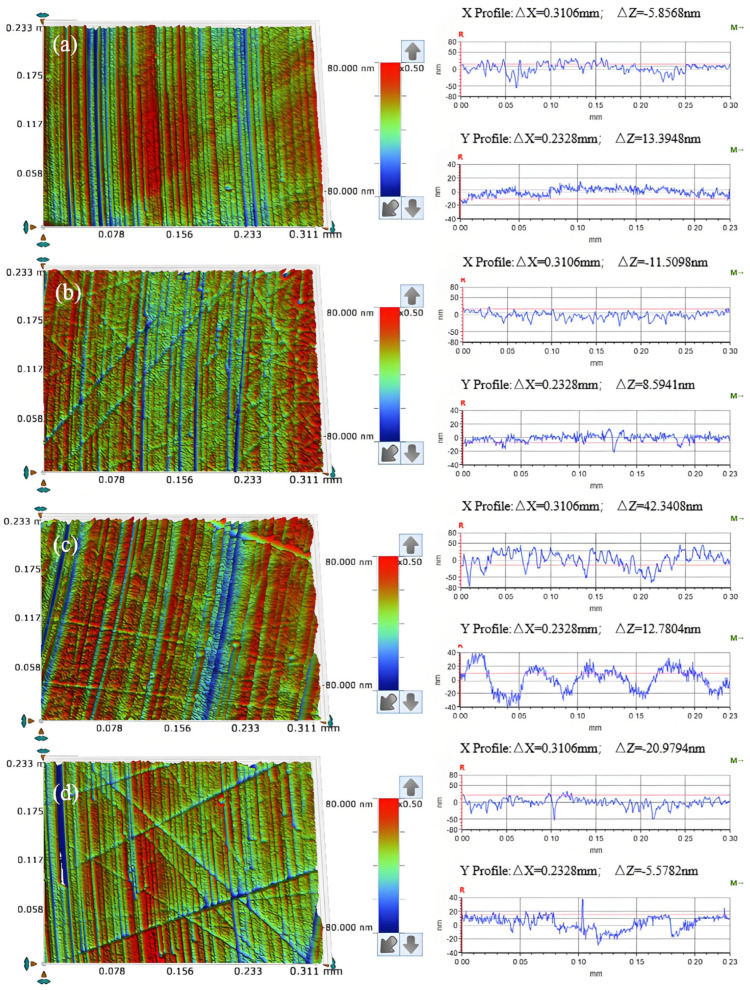
Surface morphology (**left**) and cross-section curves (**right**) of friction-worn workpieces under different polishing pad conditions (**a**) SH-Q13K-600; (**b**) SH-Q13K-800; (**c**) SH-181K; (**d**) SH-Q20.

When employing the SH-Q13K-600 polishing pad, the COF increases rapidly within a short duration before gradually rising further. As depicted in Figure 17a, regular scratches are observed on the workpiece surface, and the cross-sectional curve exhibits relatively smooth variations.

In contrast, when using the SH-Q13K-800 polishing pad, the COF demonstrates an initial rapid increase to a certain value followed by a gradual rise, peaking at six minutes before stabilizing. Figure 17b shows that while regular grooves are present on the workpiece surface, local fluctuations in the cross-sectional curve become pronounced alongside chaotic scratches.

Concerning the SH-181K polishing pad, COF reaches a stable value shortly after initiation; as illustrated in Figure 17c, regular scratches appear on the workpiece surface; however, groove depths vary significantly and local fluctuations in the cross-sectional curve are quite severe.

Finally, when utilizing the SH-Q20 polishing pad, COF experiences a rapid initial increase followed by gradual growth. Regular scratches can be seen on this workpiece surface as well; nonetheless, significant local fluctuations and chaotic deep scratches characterize its cross-sectional curve as shown in Figure 17d.

**Figure 18:**
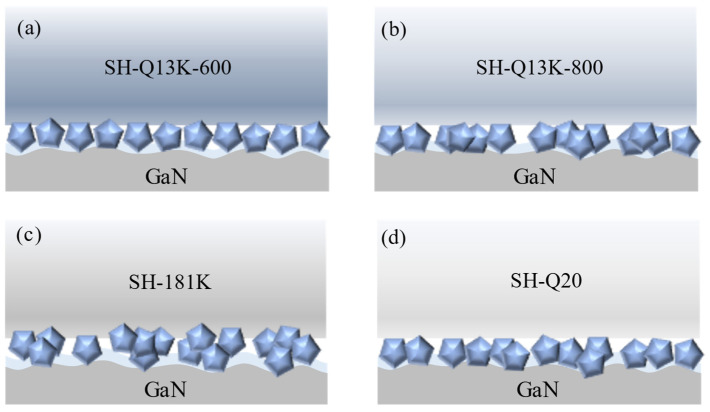
Material removal behavior under different polishing pad conditions (**a**) SH-Q13K-600; (**b**) SH-Q13K-800; (**c**) SH-181K; (**d**) SH-Q20.

The hardness, density, and compression ratio of the polishing pad are the critical factors influencing material removal during the polishing process. A lower hardness and density facilitate easier embedding of the abrasive. At the same time, an appropriate compression ratio enhances resistance to deformation throughout the polishing procedure, leading to a uniform and consistent pattern of material removal. As illustrated in Figure 18a, the SH-Q13K-600 polishing pad exhibits lower hardness and relatively reduced density, allowing for better embedding of abrasives during experimentation and more effective removal of the oxide layer on the workpiece surface. This results in a regular scratch pattern with consistent depth on the workpiece surface. Conversely, as shown in Figure 18b, the SH-Q13K-800 polishing pad possesses higher hardness and density, resulting in fewer embedded abrasives within it. On the one hand, the abrasive that can effectively act on the oxide layer on the surface of the workpiece decreases, meaning that the abrasive’s ability to remove material from the surface of the workpiece decreases; on the other hand, the increased free abrasive in the system makes the three-body wear between the abrasive and the workpiece, resulting in messy scratches on the surface of the workpiece. As shown in Figure 18c, the relatively low hardness and density of the SH-181K polishing pad increased the number and depth of abrasive embedded in the polishing process, which improved the clamping strength of the pad on the abrasive and achieved the removal of material from the surface of the workpiece. However, due to the hardness and compression of the polishing pad being relatively low, resistance to deformation is relatively poor, and the fit state with the workpiece is not stable enough; alongside other factors, this results in friction abrasion on the surface of the workpiece and scratches of different depths. While the SH-Q20 polishing pad has a greater hardness and density, the difficulty of embedding the abrasive in the polishing pad is increased, as shown in Figure 18d. On the one hand, this will increase the amount of free abrasive and cause more three-body wear between the abrasive and the workpiece, resulting in messy scratches on the surface of the workpiece [31]; on the other hand, it means that the abrasive embedded in the depth of the oxidized layer on the surface of the workpiece increases. This scratches the surface of the workpiece while removing the oxidized layer on the surface of the workpiece, and thus the depth of the scratches is relatively large.

**Figure 19:**
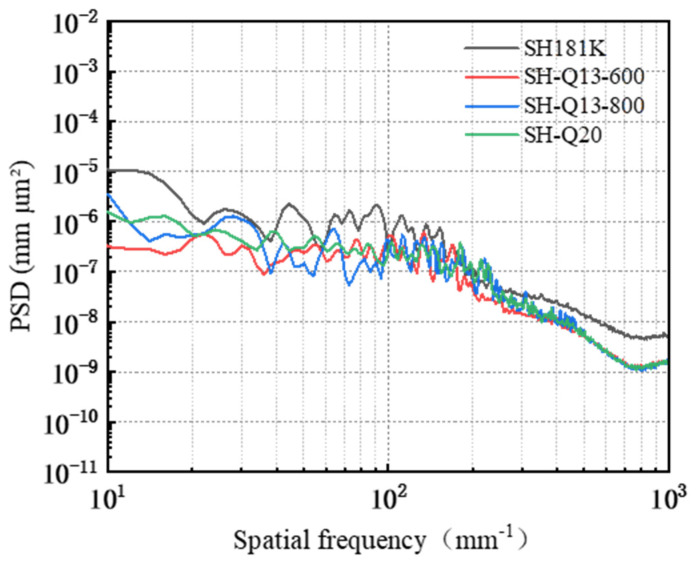
Surface power spectral density distribution under different polishing pad conditions.

A PSD analysis was conducted on the surface of the workpiece following friction and wear, with the results presented in Figure 19. In the 10 mm^−1^~36.17 mm^−1^ band, the PSD curves corresponding to the three polishing pads SH-Q13K-600, SH-Q13K-800, and SH-Q20 cross each other but do not overlap at all; compared with these three pads, the PSD curve of the surface of the workpieces corresponding to the SH-181K polishing pad show obvious grading, and its PSD values are the largest. In the frequency range from 36.17 mm^−1^ to 96.44 mm^−1^, the PSD curves for all four polishing pads intersected, with their values ranked in descending order as follows: SH-181K > SH-Q13K-800 > SH-Q20 > SH-Q13K-600. Within the frequency range from 96.44 mm^−1^ to 337.55 mm^−1^, these curves not only overlapped but also partially coincided, leading to a gradual reduction in the differences among them during this interval. Beyond 337.55 mm^−1^, all four abrasives’ PSD curves displayed a slow decline and began to level off, with those corresponding to SH-Q13K-600, SH-Q13K-800, and SH-Q20 converging more closely together. Compared to these three types of polishing pads, the PSD value associated with the SH-181K polishing pad was notably higher; thus, within this frequency range, it is evident that the workpiece surface morphologies produced by SH-Q13K-600, SH-Q13K-800, and SH-Q20 were similar and superior to that generated by the SH-181K polishing pad.

Integrating the surface morphology images, cross-sectional profiles, and COF analysis results obtained from the friction and wear experiments, it can be inferred that the effectiveness of material removal across different polishing pads is ranked as follows: SH-Q13K-600 > SH-181K > SH-Q13K-800 > SH-Q20. Furthermore, the workpiece surface quality is ordered from highest to lowest as follows: SH-Q13K-600, SH-Q20, SH-Q13K-800, and SH-181K.

### 3.5. Effect of Polishing Pressure on the Removal Behavior of Single-Crystal GaN Materials

In the electro-Fenton chemical mechanical polishing system, friction wear experiments were performed on the workpiece surface utilizing a Pin–Disk abrasion setup under varying applied pressures (0.0641 MPa, 0.1281 MPa, 0.2242 MPa, and 0.2883 MPa) to investigate the effect of pressure on both the material removal capability of the workpiece surface and the facilitation of oxide layer formation.

**Figure 20:**
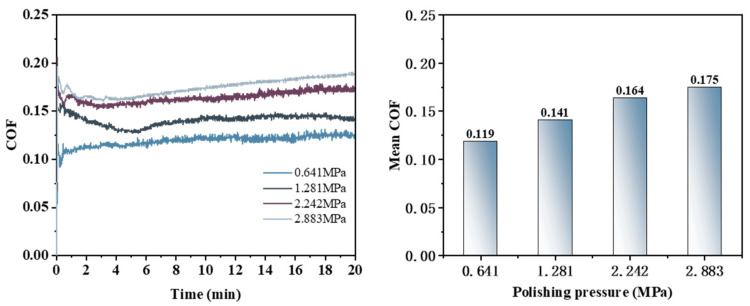
Friction coefficient curve and average friction coefficient under different polishing pressure conditions.

As illustrated in Figure 20, the behavior of the friction coefficient curve during the Pin–Disk friction and wear experiment varies with increasing polishing pressure. The average friction coefficients, ranked from highest to lowest, are as follows: 0.175 (0.2883 MPa) > 0.164 (0.2242 MPa) > 0.141 (0.1281 MPa) > 0.119 (0.0641 MPa). This indicates that the average COF increases progressively with rising polishing pressure.

At a polishing pressure of 0.641 MPa, the COF exhibits a rapid increase within a short duration before stabilizing. When the polishing pressure is elevated to 0.1281 MPa, the COF initially experiences a brief rise, reaching a peak, followed by a significant decrease and then a slight rebound before stabilization. At 0.2242 MPa, the COF similarly ascends quickly to its peak but subsequently undergoes a minor decline before ultimately stabilizing. In contrast, at 0.2883 MPa, the COF first attains its peak value, then slightly decreases before demonstrating an ongoing upward trend.

**Figure 21:**
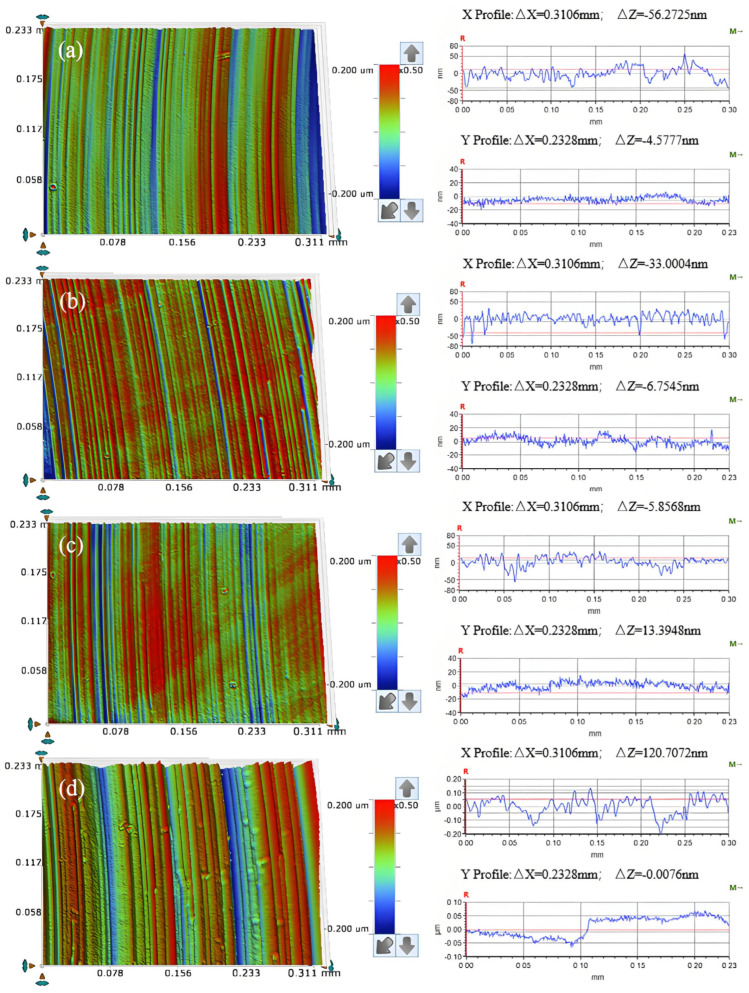
Surface morphology (**left**) and section curve (**right**) of the friction and wear on workpieces under different polishing pressures (**a**) 0.0641 MPa; (**b**) 0.1281 MPa; (**c**) 0.2242 MPa; (**d**) 0.2883 MPa.

As can be seen from Figure 21a, regular scratches appear on the surface of the workpiece after friction abrasion, and the change in the cross-section curve is relatively smooth. As shown in Figure 21b, the workpiece surface shows uniform and regular scratches, and its cross-section curve is relatively smooth, and compared with that of the cross-section curve for a polishing pressure of 0.0641 MPa, there is a significant improvement in the direction perpendicular to the regular scratches (X Profile). As shown in Figure 21c, the surface of the workpiece shows regular scratches, and the cross-section profile along the direction perpendicular to the regular scratches (X Profile) is smoother compared with the cross-section profile for a polishing pressure of 0.1281 MPa, but the depth of material removal on the surface of the workpiece fluctuates slightly along the direction of the regular scratches (Y Profile). As shown in Figure 21d, there are regular scratches on the surface of the workpiece, but the depth of the scratches varies and there are obvious pits on the surface of the workpiece, with significant fluctuations in the cross-sectional profile.

**Figure 22:**
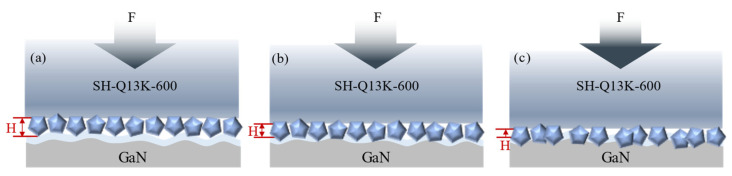
Material removal behavior under different polishing pressure conditions (**a**) the polishing pressure is too low; (**b**) the polishing pressure is moderate; (**c**) excessive polishing pressure.

As illustrated in Figure 22, the level of polishing pressure exerts a direct influence on the depth of abrasive embedded into the workpiece surface. In Figure 22a, it is evident that lower polishing pressures result in shallower embedding depths of abrasives, which diminishes their effectiveness in removing the oxide layer and consequently slows both the material removal rates and oxide layer generation rates. Conversely, as polishing pressure increases, the force exerted by the abrasives on the workpiece surface intensifies, facilitating deeper embedding into the oxide layer (Figure 22b), thereby significantly enhancing oxide layer removal capabilities. Once the original oxide layer has been eliminated, this process promotes both the formation and subsequent removal of a new oxide layer while effectively increasing material removal from the workpiece surface [32]; at this stage, scratch depth tends to stabilize. However, excessive polishing pressure can lead to a significant increase in the degree of abrasive embedded into the oxide layer on the workpiece surface, as shown in Figure 22c. This not only enhances the material removal effect but also results in a significant increase in the depth of scratches on the polished surface.

**Figure 23:**
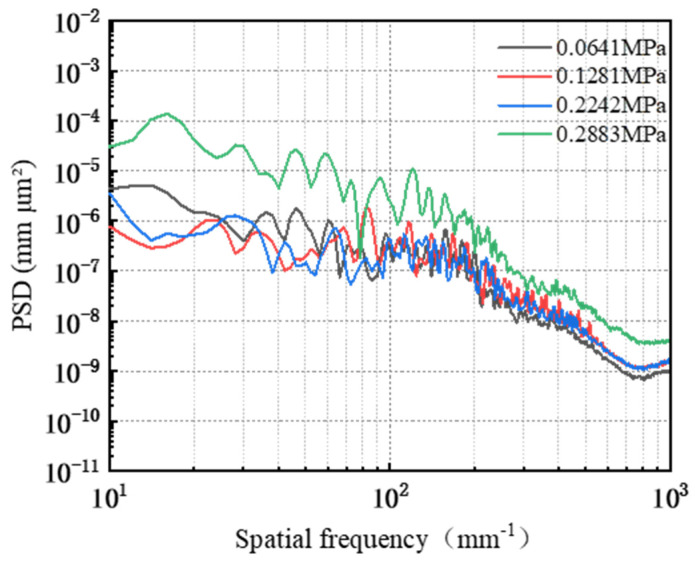
Surface power spectral density distribution under different polishing pressure conditions.

The power spectral density PSD analysis was carried out on the surface of the workpiece after friction wear, and the results are shown in Figure 23. In the 10 mm^−1^~76.35 mm^−1^ band, the PSD curves corresponding to a polishing pressure of 0.0641 MPa, 0.1281 MPa, and 0.2242 MPa cross but do not overlap, and the PSD value corresponding to a polishing pressure of 0.2883 MPa is significantly larger compared with the PSD values corresponding to the three polishing pressures mentioned above. The PSD curves corresponding to the four abrasive concentrations crossed but did not overlap in the 76.35 mm^−1^~552.56 mm^−1^ band. Among them, the PSD value corresponding to a polishing pressure of 0.2883 MPa is the largest, and the PSD difference corresponding to the other three polishing pressures is gradually diminished. That is, in this band, the workpiece surface roughness Ra corresponding to 0.2883 MPa is the largest, and the difference between the workpiece surface morphology corresponding to the other three polishing pressures is becoming increasingly smaller. In the frequency band following 552.56 mm^−1^, the PSD curves corresponding to the four polishing pressures have the same trend of change, all of them show a slow decline and tend to level off. The PSD values corresponding to different polishing pressures are in the following order: 0.2883 MPa > 0.2242 MPa ≈ 0.1281 MPa > 0.0641 Ma. Among them, the PSD curves corresponding to the polishing pressures of 0.1281 MPa and 0.2242 MPa overlap, i.e., the surface morphology on the workpieces corresponding to the two types of polishing pressures are similar.

Integrating the surface morphology images, cross-sectional curves, and COF analysis results obtained from the friction and wear experiments, it can be concluded that the effectiveness of material removal under varying pressure conditions is ranked as follows: 0.2883 MPa > 0.2242 MPa > 0.1281 MPa > 0.0641 MPa. The surface quality of the workpiece ranks from best to worst as follows: 0.2242 MPa, 0.1281 MPa, 0.0641 MPa, and finally 0.2883 MPa.

## 4. Conclusions

In this paper, by analyzing the change rule of the COF curve in Pin–Disk friction wear, workpiece surface morphology, and PSD, we explore the influence of the abrasive type, abrasive concentration, abrasive grain size, polishing pad type, and polishing pressure on the material removal behavior as well as the promotion of the generation of the oxidized layer in the process of electro-Fenton chemical–mechanical polishing, and reach the following conclusions:

(1) Different types of abrasives exhibit different removal capabilities during electro-Fenton chemical mechanical polishing. Compared to other abrasives, diamond has significant advantages in terms of stability and material removal ability. The grain size, concentration, and polishing pressure of the abrasive modulate the removal of the oxide layer, mainly by influencing the depth of its embedding in the oxide layer on the surface of the workpiece. In addition, the type of polishing pad affects the material removal capability on the workpiece surface by changing the holding power of the abrasive and the fit between the workpiece and the polishing pad.
(2) A comprehensive examination of the workpiece surface topographies, cross-section curves, and PSD analysis results after the friction wear experiments show that the mechanical removal effect promotes the generation of the oxide layer on the surface of the workpiece best when using W0.5 diamond with a concentration of 1.5 wt% and a polyurethane pad (SH-Q13K-600) as the polishing pad, and applying a polishing pressure of 0.2242 MPa; at the same time the abrasive shows good material removal capability.
(3) In the initial phase of the cutting-disk friction and wear experiment, the abrasive’s scraping action on the workpiece surface results in pronounced scratches, leading to a rapid increase in the friction force and consequently a sharp rise in the friction coefficient. As the reaction progresses, these scratches expand the contact area with ·OH, further facilitating oxide layer formation. Simultaneously, both the abrasive and the polishing pad effectively remove the existing oxide layers from the workpiece surface while accelerating the development of new oxide layers. When there is a balance between the generation rate and the removal rate of the oxide layer on the workpiece surface, material removal stabilizes, resulting in the gradual stabilization observed in the COF curve.

## Figures and Tables

**Table 1:** Parameters of the electro-Fenton solution and friction and wear test conditions.

Factors	Levels	Remarks
Base solution	0.1 mol/L Na_2_SO_4_	Basic parameters of electro-Fenton solution preparation
Electro-Fenton catalyst	3 wt% Fe-C	
H_2_O_2_	Start with 5 wt% and refill at 0.3 g/min	
pH	3	
Pin	Polishing pad φ3 mm	Pin–Disk type simulated CMP friction base test conditions
Disk	10 × 10 mm^2^ GaN-Ga surface	
Friction radius	2 mm	
Rotational speed	150 rpm	
The current intensity	20 mA	
Time	20 min	

**Table 2:** Experimental design of Pin–Disk simulated CMP friction and wear.

Serial Number	Factors				
	Type of Abrasive	Abrasive Concentration (wt%)	Abrasive Particle Size	Type of Polishing Pad	Polishing Pressure (MPa)
A1	Diamond	5	W1 (≈0.7 μm)	SH-Q13K-600	0.2242
A2	Alumina	5	W1	SH-Q13K-600	0.2242
A3	Silicon carbide	5	W1	SH-Q13K-600	0.2242
A4	Silica Sol	5	120 nm	SH-Q13K-600	0.2242
A5	Diamond	0.5	W1	SH-Q13K-600	0.2242
A6	Diamond	1	W1	SH-Q13K-600	0.2242
A7	Diamond	1.5	W1	SH-Q13K-600	0.2242
A8	Diamond	2	W1	SH-Q13K-600	0.2242
A9	Diamond	1	W0.2 (≈0.2 μm)	SH-Q13K-600	0.2242
A10	Diamond	1	W0.5 (≈0.5 μm)	SH-Q13K-600	0.2242
A11	Diamond	1	W1.5 (≈1.5 μm)	SH-Q13K-600	0.2242
A12	Diamond	1	W0.5	SH-Q13K-800	0.2242
A13	Diamond	1	W0.5	SH-Q20	0.2242
A14	Diamond	1	W0.5	SH-181K	0.2242
A15	Diamond	1	W0.5	SH-Q13K-600	0.0641
A16	Diamond	1	W0.5	SH-Q13K-600	0.1281
A17	Diamond	1	W0.5	SH-Q13K-600	0.2883

**Table 3:** Polishing pad types and related parameters.

Model Number	Hardness (JIS-A)	Density (g/cm^3^)	Compression Ratio	Thickness (mm)
SH-Q13K-600	78	0.47	6.3	1.4
SH-Q13K-800	90	0.53	5.5	1.4
SH-181K	70	0.49	5.8	1.4
SH-Q20	90	0.56	6.7	1.4

## Data Availability

The original contributions presented in this study are included in the article. Further inquiries can be directed to the corresponding author.
